# Food Additive Solvents Increase the Dispersion, Solubility, and Cytotoxicity of ZnO Nanoparticles

**DOI:** 10.3390/nano13182573

**Published:** 2023-09-17

**Authors:** Hye-In Lee, Ri-Ye Kwon, Soo-Jin Choi

**Affiliations:** Division of Applied Food System, Major of Food Science & Technology, Seoul Women’s University, Seoul 01797, Republic of Korea; 2018111058@swu.ac.kr (H.-I.L.); mystic2121@swu.ac.kr (R.-Y.K.)

**Keywords:** zinc oxide, food additive solvents, interactions, solubility, hydrodynamic diameters, cytotoxicity

## Abstract

Zinc oxide (ZnO) nanoparticles (NPs) are utilized as a zinc (Zn) fortifier in processed foods where diverse food additives can be present. Among them, additive solvents may strongly interact with ZnO NPs by changing the dispersion stability in food matrices, which may affect physico-chemical and dissolution properties as well as the cytotoxicity of ZnO NPs. In this study, ZnO NP interactions with representative additive solvents (methanol, glycerin, and propylene glycol) were investigated by measuring the hydrodynamic diameters, solubility, and crystallinity of ZnO NPs. The effects of these interactions on cytotoxicity, cellular uptake, and intestinal transport were also evaluated in human intestinal cells and using in vitro human intestinal transport models. The results revealed that the hydrodynamic diameters of ZnO NPs in glycerin or propylene glycol, but not in methanol, were significantly reduced, which is probably related to their high dispersion and increased solubility under these conditions. These interactions also caused high cell proliferation inhibition, membrane damage, reactive oxygen (ROS) generation, cellular uptake, and intestinal transport. However, the crystal structure of ZnO NPs was not affected by the presence of additive solvents. These findings suggest that the interactions between ZnO NPs and additive solvents could increase the dispersion and solubility of ZnO NPs, consequently leading to small hydrodynamic diameters and different biological responses.

## 1. Introduction

Zinc oxide (ZnO) is utilized as a food additive to increase the nutritional value of food, acting as a zinc (Zn) fortifier. Indeed, Zn is an essential trace element that plays a role in diverse metabolic processes and biological functions [1,2,3,4]. ZnO is synthetically produced as a white powder insoluble in water. It is listed as generally recognized as safe (GRAS) by the United States (US) Food and Drug Administration (FDA), although high exposure to it can cause a flu-like illness, namely metal fume fever, with symptoms including nausea, vomiting, headache, fever, fatigue, chills, muscle aches, and weakness [4,5,6,7,8,9]. Meanwhile, Zn deficiency can lead to diarrhea, hair loss, delayed wound healing, poor appetite, and delayed growth and sexual maturation in children [2,8,9,10]. 

Although ZnO is considered a water-insoluble material, it can be highly soluble under acidic conditions where the pH ranges from two to four [11,12,13]. Indeed, many processed foods have an acidic pH rather than an alkaline pH, which may cause the release of Zn ions from ZnO particles to some extent [14,15,16]. This is an important point to address because the toxicity of ZnO was reported to be primarily associated with the toxicity of Zn ions dissolved from the particles [17,18,19]. Moreover, ZnO can be produced as nano-sized material due to the development of nanotechnology, which possess high surface area-to-volume ratio, resulting in high reactivity and different responses compared with bulk-sized materials. Recent studies demonstrated that ZnO nanoparticles (NPs) had higher solubility, toxicity, and cellular uptake than micro-sized particles [20,21,22,23], supporting the high reactivity of the former than the latter. 

When ZnO is directly added to processed foods as a food additive, ZnO interactions with food matrices might occur [16]. Several reports demonstrated that the interaction of ZnO NPs with saccharides, proteins, or polyphenols resulted in the high absorption, cellular uptake, and cytotoxicity of ZnO NPs [24,25,26,27,28]. Hence, the interactions between ZnO NPs and food components or additives should be considered when predicting potential toxicity and biological responses of ZnO directly added to food. Until now, most studies have focused on the interactions between NPs and food matrices, but little information is available about ZnO interactions with food additives. Among various food additives, methanol (methyl alcohol) is a food additive extraction solvent, used to extract or dissolve target components with a residual level of below 50 ppm in the final product [29,30,31]. Naturally produced methanol content in fruit wines, brandies, and liqueurs should be less than 500 ppm [32]. Glycerin is used as a food additive for food and beverage products, which prevents sugar crystallization and provides smoothness and sweetness, serving as a humectant, solvent, preservative, and stabilizer [33,34]. Propylene glycol is classified as a GRAS food additive by US FDA and used to absorb extra water and maintain moisture in food as a humectant, and also to improve the texture, flavor, appearance, and shelf-life of processed foods as a stabilizer and solvent carrier [35,36,37]. Glycerin and propylene glycol are generally considered safe when low levels are directly added to food [34,35,37]. These food additive solvents may interact with ZnO NPs, resulting in their dissolution and different particle or ionic fates in processed foods, which can also affect the toxicity and biological responses of ZnO NPs. 

In this study, the interactions between ZnO NPs and three representative additive solvents (methanol, glycerin, and propylene glycol) were evaluated by measuring hydrodynamic diameters, zeta potentials, and solubility, and by analyzing their crystalline phases. The interactions’ effects on the cellular toxicity, cellular uptake, and intestinal transport of ZnO NPs were also investigated in human intestinal cells and using in vitro human intestinal transport models to understand the relation between physico-chemical changes and biological responses caused by the interactions. 

## 2. Materials and Methods

### 2.1. Materials 

ZnO NPs (<100 nm), Zn standard solution, and ethylenediaminetetraacetic acid disodium salt dehydrate (EDTA) were purchased from Sigma-Aldrich (St. Louis, MO, USA). Methanol, nitric acid (HNO_3_), hydrogen peroxide (H_2_O_2_), and hydrochloride (HCl) were provided by Samchun Pure Chemical Co., Ltd. (Pyeongtaek, Republic of Korea). Glycerin and propylene glycol were obtained from Joylife Inc. (Gimhae, Gyeongsangnam-do, Republic of Korea). Minimum essential medium (MEM), Roswell Park Memorial Institute (RPMI) 1640, Dulbecco’s modified Eagle medium (DMEM), Dulbecco’s phosphate-buffered saline (DPBS), Hanks’ balanced salt solution (HBSS), heat-inactivated fetal bovine serum (FBS), penicillin, and streptomycin were supplied by Welgene Inc. (Gyeongsan, Gyeongsangbuk-do, Republic of Korea). 3-(4,5-dimethyl thiazol-2-yl)-2,5-diphenyltetrazolium bromide (MTT) and 2′,7′-dichlorofluoroescein diacetate (H_2_DCFDA) were obtained from Duchefa Biochemie (Haarlem, the Netherlands) and Molecular Probes, Inc. (Eugene, OR, USA), respectively. A CytoTox 96 Non-Radioactive Cytotoxicity Assay kit and sodium dodecyl sulfate (SDS) were provided by Promega (Madison, WI, USA) and Elpis Biotech Inc. (Daejeon, Korea), respectively. Matrigel^®^ matrix was obtained from Corning Inc. (Corning, NY, USA) and Transwell^®^ polycarbonate inserts were purchased from SPL Life Science Co., Ltd. (Pocheon, Gyeonggi-do, Republic of Korea). Lucifer Yellow CH (LY) was purchased from Biotium Inc. (San Francisco Bay, CA, USA).

ZnO NPs (500 μg/mL) were suspended in distilled and deionized water (DDW); 1% methanol, 1% glycerin, and 1% propylene glycol prepared in DDW, respectively, after stirring for 30 min followed by sonication for 15 min. For cell experiments, ZnO NPs (500 μg/mL) in 1% solutions of the three different solvents were prepared in MEM. Although the maximum residual concentration of methanol as an extract solvent and in fruit-based or fermented liquors were 50 ppm and 500 ppm, respectively, the same concentration (1%) of three additive solvents was used for comparative study.

### 2.2. Characterization

The constituent particle size and morphology of ZnO NPs were analyzed by field emission scanning electron microscopy (FE-SEM; JSM-7100F, JEOL, Tokyo, Japan). From the SEM images, more than 100 particles were randomly selected, and the average particle size and size distribution were measured using ImageJ software (version 1.53k, National Institutes of Health, Bethesda, MD, USA). Dynamic light scattering (DLS) and electrophoretic light scattering methods were applied for measuring the hydrodynamic diameters and zeta potentials of ZnO NPs (100 μg/mL) in DDW, MEM, or 1% solutions of the three different solvents prepared in DDW or MEM after dispersion for 30 min and 24 h, respectively, using a Zetasizer Nano System (Malvern Instruments, Worcestershire, UK).

The crystal structure of ZnO NPs (500 μg/mL), prepared in three additive solvents as described in Section 2.1. and completely dried in powdered forms in a dry oven at 60 °C, was analyzed by the powder X-ray diffraction (XRD) patterns using a diffractometer (SmartLab, Rigaku Co., Tokyo, Japan) with Ni-filtered CuKα radiation. The conditions were as follows: λ = 1.5418 Å, 40 kV of voltage, 40 mA of current, 5–80° of scan range with 0.02° of step size, and 3°/min of scanning rate. 

### 2.3. Solubility

The solubility of ZnO NPs (5 mg/mL) was assessed using three additive solvents prepared in DW and MEM, respectively. After the designated times (0, 0.5, 1, 6, and 24 h) at 37 °C, the solutions were centrifuged at 16,000× *g* for 15 min, and the concentrations of dissolved Zn ions from the particles in the supernatants were quantified using inductively coupled plasma-atomic emission spectroscopy (ICP-AES; JY2000 Ultrace, HORIBA Jobin Yvon, Longjumeau, France) after digestion as described in Section 2.4. 

### 2.4. Digestion and ICP-AES Analysis

For quantitative analysis of ZnO NPs, the samples were digested with 60% HNO_3_ (10 mL) at ~160 °C, followed by treatment with 34.5% H_2_O_2_ (1 mL) until the mixtures were colorless and evaporated. The total Zn concentrations in the digested samples were measured after dilution with appropriate volumes of DDW by ICP-AES (JY2000 Ultrace, HORIBA Jobin Yvon). The conditions were 1000 W of radio-frequency power and 12 L/min of plasma gas flow.

### 2.5. Cell Culture

Human intestinal epithelial Caco-2 cells were obtained from Korean Cell Line Bank (Seoul, Republic of Korea). The cells were maintained in MEM where 10% FBS, 100 units/mL of penicillin, and 100 μg/mL of streptomycin were supplemented under a 5% CO_2_ atmosphere at 37 °C.

### 2.6. Cell Proliferation

The effect of the interactions between ZnO NPs and food additive solvents (1% methanol, 1% glycerin, or 1% propylene glycol) on cell proliferation was evaluated by MTT assay. Cells (1 × 10^4^ cells/100 μL in a 96-well plate) were incubated with the designated concentrations of the samples for 24 h, followed by treatment with MTT solution (10 μL) for 4 h. The cells were then further treated with 100 μL of solubilization solution (0.01 M HCl + 10% SDS) overnight. The absorbance of the solutions at 570 nm was measured using a microplate reader (SpectraMax^®^ M3, Molecular Devices, Sunnyvale, CA, USA). Cells treated in the absence of samples were used as controls.

### 2.7. Lactate Dehydrogenase (LDH) Leakage

The effect of the interactions between ZnO NPs and food additive solvents (1% methanol, 1% glycerin, or 1% propylene glycol) on cell membrane damage was estimated using a CytoTox 96 Nonradioactive Cytotoxicity Assay kit which measures intracellular LDH release into an extracellular medium. Cells (4 × 10^4^ cells/mL in a 24-well plate) were treated with the designated concentrations of the samples for 24 h. The supernatants of the cell culture medium were then obtained after centrifugation, and 50 μL of a substrate solution was added to 50 μL of the supernatants. Further incubation was carried out in the dark at room temperature. After 30 min, 50 μL of a stop solution was added, and the absorbance of the solutions at 490 nm was measured with a microplate reader (SpectraMax^®^ M3, Molecular Devices). Cells treated in the absence of samples were used as controls.

### 2.8. Reactive Oxygen Species (ROS) Generation

The ROS induced by the interactions between ZnO NPs and food additive solvents (1% methanol, 1% glycerin, or 1% propylene glycol) was measured with a peroxide-sensitive fluorescent probe, H_2_DCFDA. Cells (1 × 10^4^ cells/100 μL in a 96-well plate) were exposed to the designated concentrations of the samples for 24 h, and further treated with 20 μM H_2_DCFDA for 30 min at 37 °C in the dark. The cells were washed with PBS and dichlorofluorescein (DCF) fluorescence (excitation at 485 nm and emission at 535 nm) and were immediately measured using a fluorescence microplate reader (SpectraMax^®^ M3, Molecular Devices). Cells treated in the absence of samples were used as controls.

### 2.9. Cellular Uptake

The cells (1 × 10^6^ cells/2 mL in a 6-well plate) were exposed to ZnO NPs (50 μg/mL in 1% solvents) for 6 h. After washing the cells with DPBS three times, 5 mM EDTA was added to the cells and incubated for 40 s to detach just-adsorbed NPs on the cell surface. The cells were harvested with a scraper after washing with DPBS three-times. After centrifugation and re-suspension in DDW, total cellular Zn levels were analyzed as described in Section 2.4. The cells treated without samples were used as controls.

### 2.10. Intestinal Transport

Microfold (M) cells are predominantly present in the follicle-associated epithelium (FAE) and are responsible for the uptake of antigens and macromolecules [38]. Thus, a FAE model was prepared as previously demonstrated [27,38]. Matrigel^®^ matrix-coated Transwell^®^ inserts were prepared in serum-free DMEM for 2 h. After washing with the serum-free medium, Caco-2 cells (1 × 10^6^ cells/well) were grown on apical sides for 14 days. The co-cultures were then made by adding lymphoma Raji B cells (1 × 10^6^ cells/well) to the basolateral sides, maintained for 5 days, and stopped when trans-epithelial electrical resistance (TEER) values ranged from 150 to 200 Ω cm^2^. ZnO NPs (50 µg/mL) prepared in DW, MEM, or 1% solutions of the three different solvents were treated on the apical sides for 6 h.

The transport of ZnO NPs by the intestinal epithelial tight junction barrier was evaluated using Caco-2 monoculture model. Caco-2 cells (4.5 × 10^5^ cells/well) were grown on apical sides for 21 days until the TEER values were more than 300 Ω cm^2^. ZnO NPs (50 µg/mL) prepared in DW, MEM, or 1% solutions of the three different solvents were treated on the apical sides for 6 h. 

The basolateral solutions obtained by the FAE and Caco-2 monolayer models were collected and the total transported Zn levels were analyzed as described in Section 2.4. The two models with an absence of ZnO NPs were used as controls. Intestinal transport (%) was calculated according to the following formula:

Intestinal transport (%) = (Zn concentration in basolateral medium − blank)/(Zn concentration treated) × 100

### 2.11. Membrane Permeability

The permeability of ZnO NPs through Caco-2 monolayer and FAE models was evaluated as previously described [39]. ZnO NPs (50 μg/mL) prepared in MEM or 1% solutions of the three different solvents were treated on the apical sides for 6 h. After incubation, the apical and basolateral parts of the models were washed with HBSS. LY solution (300 µg/mL in HBSS) was treated on the apical sides for 1 h at 37 °C and the basolateral sides were filled with HBSS. Finally, the fluorescence (excitation at 430 nm and emission at 540 nm) of the basolateral solutions was measured with a fluorescence microplate reader (SpectraMax^®^ M3, Molecular Devices). The following formula was used to calculate the permeability (%):

Permeability (%) = (basolateral medium − blank)/(LY treated − blank) × 100

### 2.12. Statistical Analysis

All results were presented as means ± standard deviations. One-way analysis of variance with Tukey’s test in SAS Ver.9.4 (SAS Institute Inc., Cary, NC, USA) was conducted to determine the significant differences among control and treated groups. Statistical significance was accepted for *p*-values of <0.05.

## 3. Results and Discussion

### 3.1. Characterization

The constituent particle size and morphology of ZnO NPs were examined using SEM. The results demonstrated that ZnO NPs had an average constituent particle size of 78.1 ± 24.6 nm with irregular shapes (Figure S1). Hydrodynamic diameters of ZnO NPs suspended in DDW were 285.0 ± 5.2 nm after dispersion for 30 min (Table 1), larger than the constituent particle sizes (Figure S1), indicating aggregate formation in aqueous solution. When the stock solution of ZnO NPs was prepared by dispersing them in three 1% additive solvents for 30 min, the hydrodynamic diameters in glycerin or propylene glycol were significantly reduced compared with those in DDW or methanol (Table 1). Meanwhile, the hydrodynamic diameters of ZnO NPs in DDW were significantly reduced after dispersion for 24 h, and more significantly reduced diameters were found when they were dispersed in glycerin or propylene glycol (Table 1). The same tendency was found when they were dispersed in 1% solvents, followed by dilution in DDW (final 0.2% solvent concentration), showing smaller hydrodynamic diameters of ZnO NPs in glycerin or propylene glycol than those in DDW or methanol (Table 1). Indeed, these solvent conditions were used for physico-chemical characterization (dispersion for 30 min, without dilution) and solubility (dispersion for 24 h, with dilution) experiments. No significant effect of solvent dilution on the hydrodynamic diameters was found, indicating that the hydrodynamic diameters of ZnO NPs were directly affected by the presence of solvents, but not by solvent concentrations. Thus, the type of solvent is an important factor for determining the hydrodynamic diameters of ZnO NPs. Moreover, it seems that glycerin and propylene glycol play roles in ZnO dispersion.

When the hydrodynamic diameters of ZnO NPs in additive solvents were prepared with/without dilution in MEM after dispersion for 30 min and 24 h, respectively, reflecting cell experimental conditions, the DLS sizes were significantly reduced only in the presence of glycerin or propylene glycol (Table 1). The smallest hydrodynamic diameters were found when ZnO NPs were dispersed in the above two additive solvents for 24 h. No dilution effect on the hydrodynamic diameters of ZnO NPs in MEM was found. All of the results suggest that additive solvents, such as glycerin and propylene glycol, can reduce hydrodynamic diameters and aggregate formation of ZnO NPs, clearly suggesting an interaction effect, whereas methanol had no effect on the DLS size, indicating the importance of solvent type in the interaction. The presence of more hydrophilic hydroxyl (-OH) groups in glycerin (three -OH) and propylene glycol (two -OH), as opposed to one -OH group in methanol, may contribute to the high dispersion of ZnO NPs under aqueous conditions [33,40,41].

The zeta potential values of ZnO NPs in DDW were 25.7 ± 1.4 mV and changed to −8.5 ± 0.3 mV in MEM (Table 1). The change in zeta potentials of ZnO NPs to negative charge in MEM seems to be attributable to diverse biological components present in MEM, which is consistent with our previous report [25]. Meanwhile, the zeta potential values of ZnO NPs did not significantly change due to the presence of three additive solvents compared with those in DDW or MEM (Table 1), indicating that the hydrodynamic diameters, but not surface charges, of ZnO NPs were affected by solvent interactions.

### 3.2. Dissolution Property

**Figure 1:**
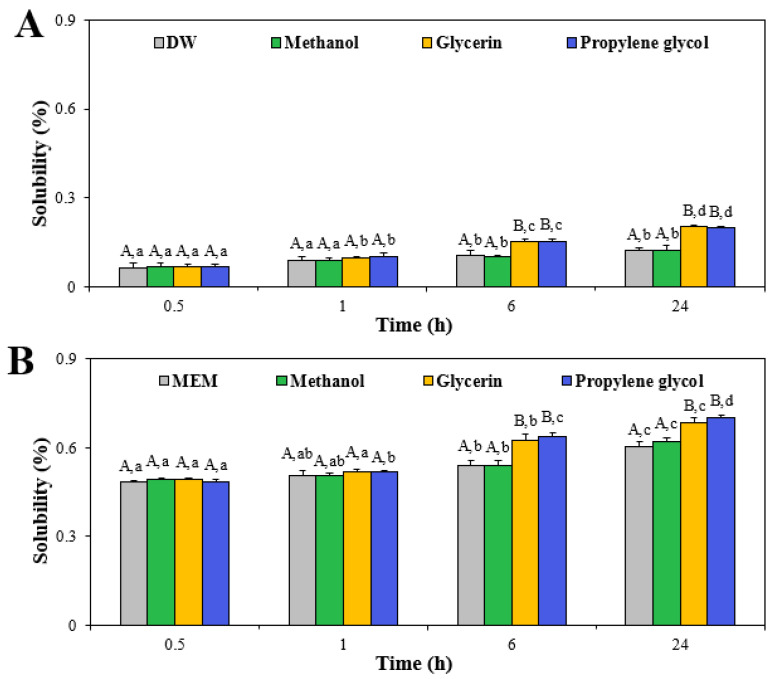
Dissolution properties of ZnO NPs when interacted with methanol, glycerin, or propylene glycol measured in (**A**) distilled water (DW) and (**B**) cell culture essential medium (MEM) at 37 °C. Different uppercase letters (A,B) indicate significant differences among ZnO NPs in DW or MEM and different solvents (*p* < 0.05). Different lowercase letters (a,b,c,d) indicate significant differences among different incubation times (*p* < 0.05).

The solubility of ZnO NPs in the presence of additive solvents was assessed since the dissolution property can be one of the most affected characteristics by solvent interactions [20,42]. It was assessed to also answer the question as to whether reduced hydrodynamic diameters of ZnO NPs in glycerin or propylene glycol were related to increased solubility. The solubility of ZnO NPs in different solvents was evaluated under two different conditions, in which they were diluted and measured in DW and MEM, reflecting physico-chemical characterization and cell experiment conditions, respectively. The results showed that the solubility of ZnO NPs significantly increased in a time-dependent manner when they were suspended in glycerin or propylene glycol regardless of dilution conditions (Figure 1). The dissolution levels of ZnO NPs in solvents diluted and measured in MEM were significantly higher than those in DW, probably associated with the interactions between ZnO NPs and the diverse biological components present in MEM. The same tendency was found in our previous report [27]. However, the solubility of ZnO NPs in solvents was less than 0.2% and 0.7% when diluted and measured in DW and MEM, respectively, indicating low solubility. The low solubility levels of ZnO NPs in DW or MEM were highly consistent with the previous report [27]. It is worth noting that the pHs of ZnO in DW, methanol, glycerin, and propylene glycol prepared in DW were 9.4, 9.0, 8.4, and 8.1, respectively, whereas their pHs in solvents prepared in MEM ranged from 8.1 to 7.9. This result indicates that the pH effect on the solubility of ZnO NPs is negligible based on their high dissolution property in acidic fluids [43,44]. It is also interesting to note that the interactions between ZnO NPs and other food components (saccharides, proteins, and polyphenols) did not remarkably affect their dissolution property [24,27,28]. Hence, these results suggest that the solubility of ZnO NPs increased in the interactions with glycerin or propylene glycol, but their overall dissolution levels were as low as less than 1%. Slightly but significantly increased solubility (~0.1%) of ZnO NPs in these two solvents may also contribute to their reduced hydrodynamic diameters to some extent, as shown in Table 1. 

### 3.3. Crystal Structure

**Figure 2:**
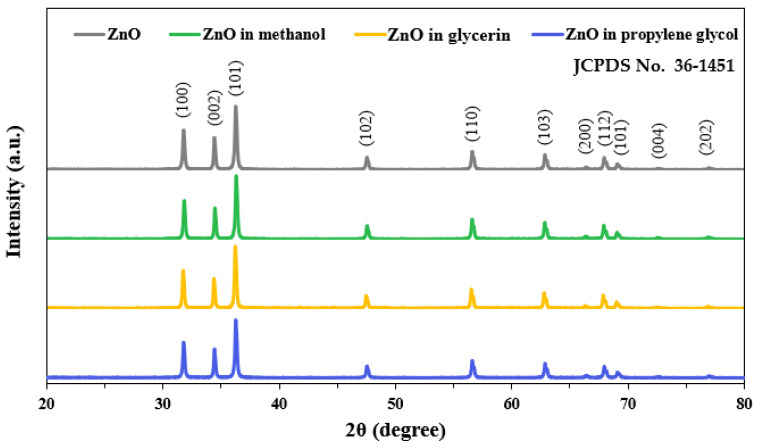
Powdered X-ray diffraction (XRD) patterns of pristine ZnO NPs and ZnO NPs when interacted with 1% different additive solvents.

The crystallinity of ZnO NPs in the presence of 1% additive solvents was analyzed by powdered XRD patterns. The results revealed that pristine ZnO NPs had typical wurtzite crystalline phases and ZnO NPs in three additive solvents had the same crystal structure (Figure 2) [45]. The crystallite sizes of ZnO, ZnO in methanol, ZnO in glycerin, and ZnO in propylene glycol based on the XRD patterns were determined to be 45.0 ± 2.8 nm, 45.4 ± 1.7 nm, 45.8 ± 1.8 nm, and 45.2 ± 2.7 nm, respectively, without significant differences among ZnO in different solvents. No significant differences in size between SEM (78.1 ± 24.6 nm) and XRD (45.0 ± 2.8 nm) results were found (*p* > 0.05), although broader size distribution was measured by SEM. Indeed, crystallite size obtained by XRD patterns provided a rough estimate of particle size, but did not always reflect a reliable estimate of particle size [46,47]. This result implies that the crystalline phase of ZnO NPs was not influenced by the interactions with solvents, although the interactions between ZnO NPs and solvents increased the dissolution levels of ZnO NPs (Figure 1). The overall low solubility (<1%) of ZnO NPs in additive solvents could explain their intact crystal structure as pristine ZnO NPs. The low solubility and intact XRD pattern results also suggest that potential toxicity of ZnO NPs in their intact particle forms should be considered for their toxicity and biological responses. 

### 3.4. Cytotoxicity

**Figure 3:**
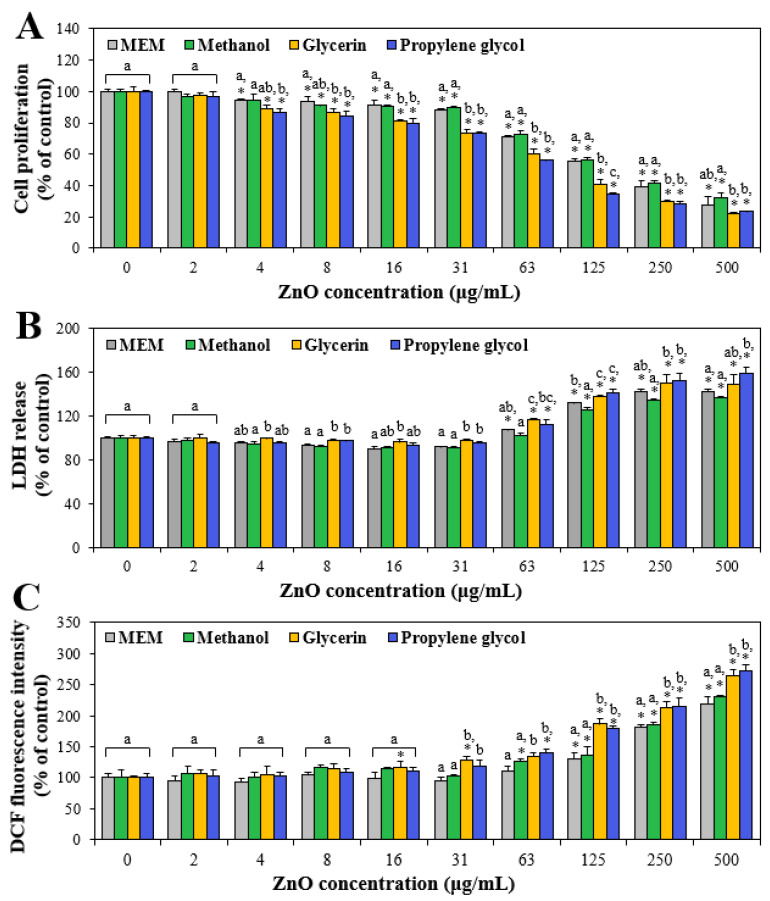
Effects of ZnO NPs interacted with methanol, glycerin, or propylene glycol on (**A**) cell proliferation inhibition, (**B**) lactate dehydrogenase (LDH) release, and (**C**) reactive oxygen species (ROS) generation in Caco-2 cells after exposure for 24 h compared with ZnO NPs in minimum essential medium (MEM). Different lowercase letters (a,b,c) indicate significant differences among ZnO NPs in MEM and different additive solvents (*p* < 0.05). * Significant differences compared with non-treated controls (*p* < 0.05).

The effects of the interactions between ZnO NPs and additive solvents on cytotoxicity were evaluated in terms of cell proliferation inhibition, membrane integrity and damage, and ROS induction in human intestinal Caco-2 cells. Figure 3A shows that cell proliferation decreased as ZnO concentrations increased. In particular, cell proliferation was more significantly inhibited by ZnO NPs dispersed in glycerin or propylene glycol at more than 4 μg/mL than by ZnO NPs in MEM or methanol, showing the interaction effect on cell proliferation inhibition. Optical microscopic images of cells treated with ZnO NPs in MEM or solvents showed that the cell number decreased as ZnO concentration increased, and more significantly decreased cell numbers were observed in the presence of glycerin or propylene glycol (Figure S2). The half-maximal inhibitory concentration (IC_50_) values of ZnO, ZnO in methanol, ZnO in glycerin, and ZnO in propylene glycol were 170.37 ± 10.83 μg/mL, 177.36 ± 10.85 μg/mL, 94.79 ± 8.20 μg/mL, and 79.90 ± 1.70 μg/mL, respectively, supporting the high toxicity of ZnO in glycerin or propylene glycol. The same tendency was obtained by the LDH release assay (Figure 3B), which measures the released levels of a stable cytosolic enzyme LDH into the extracellular medium when the membrane integrity is damaged. The result demonstrated that LDH release increased at more than 4 μg/mL, and that significantly higher LDH leakage was induced by ZnO NPs in glycerin or propylene glycol than by ZnO NPs in MEM or methanol. Intracellular ROS was also generated and increased by ZnO NPs in additive solvents in a dose-dependent manner, showing significantly high ROS induction in the presence of glycerin or propylene glycol (Figure 3C). All cytotoxicity results indicate that ZnO NPs caused cell proliferation inhibition, membrane damage, and ROS generation, as demonstrated in other previous studies [27,28,48]. The cytotoxicity increased when ZnO NPs were dispersed in the presence of glycerin or propylene glycol, but not in methanol. The high cytotoxicity of ZnO NPs in glycerin or propylene glycol could be attributed to their small hydrodynamic diameters (Table 1) and increased solubility (Figure 2) under these conditions. Indeed, it was reported that ZnO NPs exhibited higher cytotoxicity than micro-sized ZnO, and their cytotoxicity was primarily related to released Zn ions from the particles [18,21,49,50]. Further extensive study is necessary and the effect of the interaction mechanism on the toxicity of ZnO NPs in solvents is required to be elucidated.

### 3.5. Cellular Uptake

**Figure 4:**
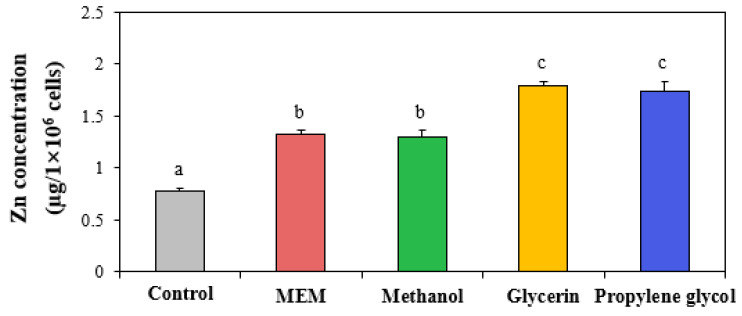
Cellular uptake of ZnO NPs when interacted with methanol, glycerin, or propylene glycol compared with ZnO NPs in minimum essential medium (MEM). Different lowercase letters (a,b,c) indicate significant differences among ZnO NPs in MEM and different additive solvents (*p* < 0.05).

The cellular uptake levels of ZnO NPs in three different solvents were evaluated by measuring the total cellular Zn concentrations using ICP-AES. The results showed that the cellular uptake of ZnO NPs significantly increased when they were prepared in glycerin or propylene glycol, whereas ZnO NPs in methanol had similar uptake compared with ZnO NPs in MEM (Figure 4). This result clearly indicates that the interactions between ZnO NPs and additive solvents can enhance the cellular uptake of ZnO NPs. This high cellular uptake of ZnO NPs in glycerin or propylene glycol compared with that in MEM or methanol could be explained by the small hydrodynamic diameters in glycerin or propylene glycol (Table 1). Many researchers reported that small-sized particles can be more easily internalized on a larger scale into cells than larger-sized particles [51,52,53]. On the other hand, the high cytotoxicity of ZnO NPs in glycerin or propylene glycol (Figure 3) seems to be also attributable to high cellular internalization amounts. 

### 3.6. Intestinal Transport

**Figure 5:**
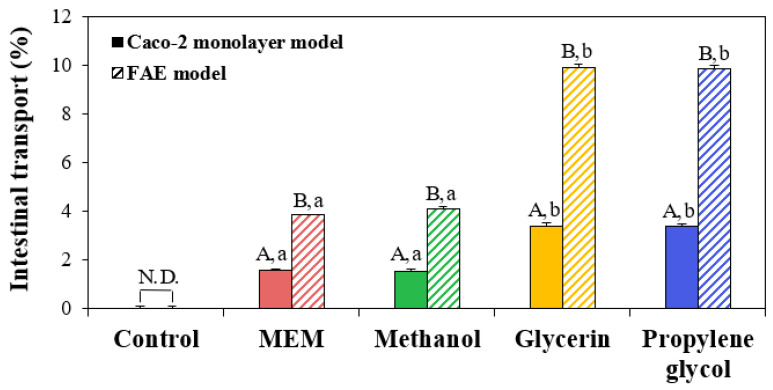
Intestinal transport of ZnO NPs when interacted with methanol, glycerin, or propylene glycol, compared with ZnO NPs in minimum essential medium (MEM), using in vitro Caco-2 monolayer and human follicle-associated epithelium (FAE) models, respectively. Different uppercase letters (A,B) indicate significant differences between Caco-2 monolayer and FAE models (*p* < 0.05). Different lowercase letters (a,b) indicate significant differences among ZnO NPs in MEM and different additive solvents (*p* < 0.05). Abbreviations: N.D., not detectable.

The interaction effects on intestinal transports of ZnO NPs were evaluated using two different in vitro intestinal epithelium models: Caco-2 monolayer and FAE models. Figure 5 shows that the amounts of intestinally transported ZnO NPs in the presence of glycerin or propylene glycol significantly increased in both Caco-2 monolayer and FAE models. Increased transport of ZnO NPs by M cells than by Caco-2 monolayer was also found, implying a major role of M cells in ZnO NP transport. These results are in good agreement with the cellular uptake results (Figure 4). Significantly higher transport of ZnO NPs by M cell than by Caco-2 monolayer was also demonstrated in the previous report [27]. The reduced hydrodynamic diameters of ZnO NPs in glycerin or propylene glycol (Table 1) could contribute to the enhanced cellular uptake (Figure 4) and intestinal transport of ZnO NPs (Figure 5) and increase cytotoxicity of ZnO NPs (Figure 3). It is worth noting that their reduced DLS sizes could be also related to their increased solubility to some extent (Figure 1). Meanwhile, ZnO NPs were reported to be internalized into cells via endocytosis, an active transport pathway [16,19]. Taken together, it is, therefore, concluded that the interactions between ZnO NPs and additive solvents can increase the even dispersion and solubility of ZnO NPs, which reduces their hydrodynamic diameters, consequently causing high cellular toxicity, cellular uptake, and intestinal transportation. 

### 3.7. Membrane Permeability

**Figure 6:**
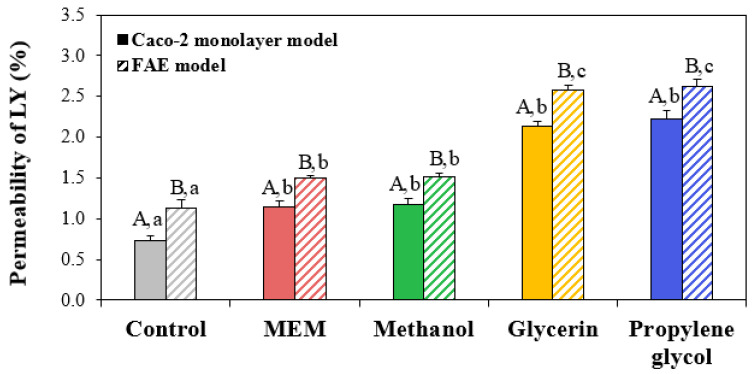
Permeability (%) of Lucifer Yellow (LY) using in vitro Caco-2 monolayer and human follicle-associated epithelium (FAE) models where ZnO NPs interacted with methanol, glycerin, or propylene glycol are compared with ZnO NPs in minimum essential medium (MEM), respectively. Different uppercase letters (A,B) indicate significant differences between Caco-2 monolayer and FAE models (*p* < 0.05). Different lowercase letters (a,b,c) indicate significant differences among ZnO NPs in MEM and different additive solvents (*p* < 0.05).

LY is a membrane-impermeable fluorescent dye, thereby serving as a quantitative marker of the cell membrane permeability [54]. The permeability can be described as the passive diffusion of particles through membranes [55]. Hence, elevated levels of ZnO NPs measured by LY using in vitro human intestinal epithelial models can indicate the amounts that have passively permeated. Figure 6 shows that the permeability of Caco-2 monolayer and FAE models without ZnO NPs were ~1%, indicating that both 2D and 3D intestinal models were well established [56]. On the other hand, the permeability of ZnO NPs in MEM and methanol increased to 1.2% and 1.5% in Caco-2 monolayer and FAE models, respectively. A high permeability of LY was found when ZnO NPs were interacted with glycerin (~2.1%) or propylene glycol (~2.6%). These results suggest that the passive diffusion of ZnO NPs was involved in their intestinal permeability to some extent, but this also increased in the presence of glycerin or propylene glycol. However, permeated amounts of ZnO NPs assessed by LY, i.e., passive diffusion, were much lower than their active transport amounts (a total of ~13% in glycerin and propylene glycol in both Caco-2 monolayer and FAE models) measured by ICP-AES (Figure 5), suggesting that active transport is the main transport pathway of ZnO NPs. Meanwhile, the highest permeability of LY was less than 3% in all cases, implying that ZnO NPs in additive solvents did not significantly affect the intestinal tight junction integrity [56]. Taken together, all of the results suggest that active and passive transportation of ZnO NPs could be increased by interaction with additive solvents. 

## 4. Conclusions

The effects of the interactions between food additive ZnO NPs and representative additive solvents (methanol, glycerin, and propylene glycol) on the physico-chemical characteristics and biological responses of ZnO NPs were investigated. The results demonstrated that the hydrodynamic diameters of ZnO NPs significantly decreased and their solubility increased when they were interacted with glycerin and propylene glycol, but not with methanol. However, the interactions did not affect the crystal structure of ZnO NPs. The interactions also caused high cytotoxicity, in terms of cell proliferation inhibition, membrane damage, and ROS generation, and enhanced cellular uptake and both passive and active transports of ZnO NPs. Taken together, these findings suggest that the interaction between ZnO NPs and additive solvents could increase the even dispersion and solubility of ZnO NPs, subsequently leading to small hydrodynamic diameters and different biological responses compared with pristine ZnO NPs. More extensive and mechanistic study is required to ascertain the interaction effect on the safety of food additive ZnO NPs.

## Figures and Tables

**Table 1:** Hydrodynamic diameters and zeta potentials of ZnO NPs in 1% different additive solvents.

Dispersant	Hydrodynamic Diameters (nm)				Zeta Potential (mV)	
	Dispersion for 30 Min		Dispersion for 24 h		Dispersion for 30 Min	
	Without Dilution	Dilution in DW or MEM	Without Dilution	Dilution in DW or MEM	Without Dilution	Dilution in DW or MEM
DDW	285.0 ± 5.2 ^a^		261.0 ± 0.5 ^b^		25.7 ± 1.4 ^a^	
Methanol in DDW	284.6 ± 7.4 ^a^	282.6 ± 11.9 ^a^	260.9 ± 0.4 ^b^	261.1 ± 0.4 ^b^	24.9 ± 0.6 ^a^	25.0 ± 0.4 ^a^
Glycerin in DDW	246.7 ± 13.8 ^b^	246.4 ± 5.8 ^b^	224.2 ± 1.0 ^c^	223.2 ± 0.8 ^c^	25.6 ± 0.3 ^a^	25.4 ± 0.4 ^a^
Propylene glycol in DDW	247.3 ± 14.4 ^b^	247.3 ± 7.3 ^b^	224.3 ± 0.6 ^c^	223.9 ± 0.8 ^c^	25.5 ± 0.3 ^a^	25.3 ± 0.5 ^a^
MEM	246.2 ± 3.0 ^a^		230.4 ± 1.2 ^bc^		8.5 ± 0.3 ^a^	
Methanol in MEM	247.4 ± 1.2 ^a^	248.0 ± 1.0 ^a^	229.5 ± 2.5 ^bc^	230.2 ± 1.9 ^c^	−8.5 ± 0.4 ^a^	−8.4 ± 0.2 ^a^
Glycerin in MEM	234.4 ± 2.1 ^bc^	233.1 ± 0.4 ^bc^	213.7 ± 2.0 ^d^	210.6 ± 1.9 ^d^	−8.6 ± 0.5 ^a^	−8.5 ± 0.2 ^a^
Propylene glycol in MEM	235.0 ± 2.0 ^bc^	236.1 ± 3.5 ^b^	213.4 ± 2.3 ^d^	213.0 ± 2.1 ^d^	−8.5 ± 0.4 ^a^	−8.5 ± 0.2 ^a^

Different lowercase letters (^a,b,c,d^) indicate significant differences among pristine ZnO NPs and ZnO NPs dispersed in different solvents in DDW or MEM (*p* < 0.05). No significant differences between with and without dilution were found (*p* > 0.05). Abbreviations: DDW, distilled deionized water; MEM, minimum essential medium.

## Data Availability

The data presented in this study are available in the article and Supplementary Materials.

## Supplementary Materials

The following are available online at https://www.mdpi.com/article/10.3390/nano13182573/s1, Figure S1: Scanning electron microscopy (SEM) image and constituent particle size distribution of ZnO NPs. Figure S2: Optical microscopic images of Caco-2 cells treated with ZnO NPs in minimum essential medium (MEM) or different additive solvents after 24 h.
