# Supplementary material for: Food Additive Solvents Increase the Dispersion, Solubility, and Cytotoxicity of ZnO Nanoparticles
Source: Nanomaterials (Basel). 2023 Sep 17;13(18):2573. doi: 10.3390/nano13182573 (PMC10534380; doi:10.3390/nano13182573)
Supplement: Supplementary file 1 [file nanomaterials-13-02573-s001.zip › nanomaterials-2606263-supplementary.pdf]

Supplementary Materials

Food Additive Solvents Increase the Dispersion, Solubility, and Cytotoxicity of ZnO Nanoparticles

Hye-In Lee, Ri-Ye Kwon and Soo-Jin Choi *

Division of Applied Food System, Major of Food Science & Technology, Seoul Women's University, Seoul 01797, Republic of Korea; 2018111058@swu.ac.kr (H.-I.L.); mystic2121@swu.ac.kr (R.-Y.K.)

* Correspondence: sjchoi@swu.ac.kr; Tel.: +82-2-970-5634

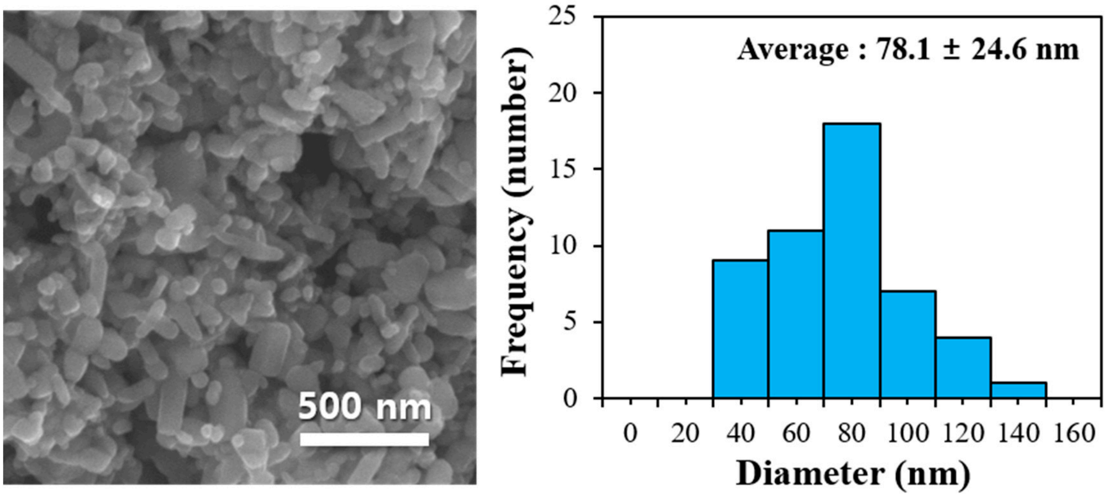

Figure S1. Scanning electron microscopy (SEM) image and size distribution of ZnO NPs. Particle size distribution was determined by randomly selecting 100 particles from SEM images.

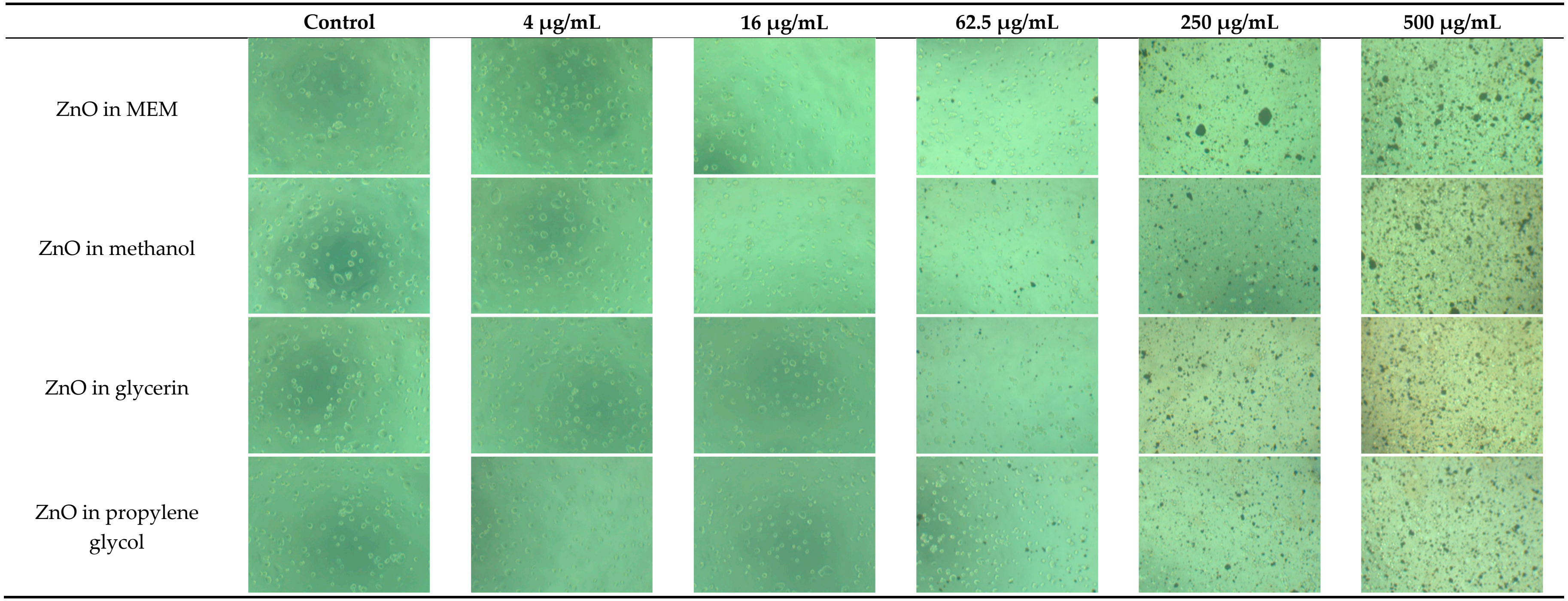

Figure S2. Optical microscopic images of Caco-2 cells treated with ZnO NPs in minimum essential medium (MEM) or different additive solvents after 24 h. .
